# EDTA a Novel Inducer of Pisatin, a Phytoalexin Indicator of the Non-Host Resistance in Peas

**DOI:** 10.3390/molecules20010024

**Published:** 2014-12-23

**Authors:** Lee A. Hadwiger, Kiwamu Tanaka

**Affiliations:** Department of Plant Pathology, Washington State University, Pullman, WA 99164-6430, USA; E-Mail: kiwamu.tanaka@wsu.edu

**Keywords:** DNA damage, pathogenesis-related genes, defensins, DNA-specific, disease resistance

## Abstract

Pea pod endocarp suppresses the growth of an inappropriate fungus or non-pathogen by generating a “non-host resistance response” that completely suppresses growth of the challenging fungus within 6 h. Most of the components of this resistance response including pisatin production can be elicited by an extensive number of both biotic and abiotic inducers. Thus this phytoalexin serves as an indicator to be used in evaluating the chemical properties of inducers that can initiate the resistance response. Many of the pisatin inducers are reported to interact with DNA and potentially cause DNA damage. Here we propose that EDTA (ethylenediaminetetraacetic acid) is an elicitor to evoke non-host resistance in plants. EDTA is manufactured as a chelating agent, however at low concentration it is a strong elicitor, inducing the phytoalexin pisatin, cellular DNA damage and defense-responsive genes. It is capable of activating complete resistance in peas against a pea pathogen. Since there is also an accompanying fragmentation of pea DNA and alteration in the size of pea nuclei, the potential biochemical insult as a metal chelator may not be its primary action. The potential effects of EDTA on the structure of DNA within pea chromatin may assist the transcription of plant defense genes.

## 1. Introduction

**Figure 1:**
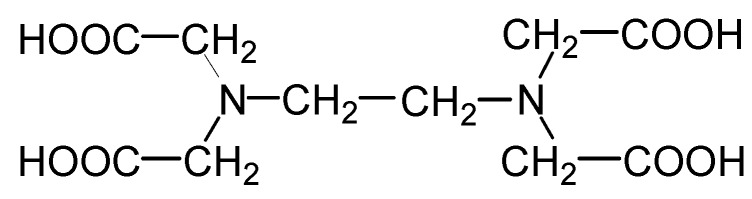
Structural formula of EDTA (ethylenediaminetetraacetic acid).

The pea endocarp system [1] has been employed to follow and understand the transcription initiation of the nonhost resistance response in plants at chromatin sites targeted by DNA-specific gene activators, one of which is a fungal DNase [2,3]. Some of the components that elicit phytoalexin production may also act without directly targeting DNA [4]. The compound EDTA (Figure 1), used traditionally in biochemistry as a chelating agent [5], was not expected to target cellular DNA or induce the phytoalexin pisatin.

FsphDNase is a natural biotic elicitor of both pisatin and the nonhost disease resistance response [3]. It is released from *Fusarium solani* f.sp. *phaseoli* (Fsph), a pathogen of bean, and causes single strand nicks in DNA in temporal association with the induction of pisatin [3]. This fungal enzyme’s catalytic action is dependent on Mn^2+^ [3]. EDTA was employed in the pea/Fsph interaction with the intention to chelate metal cofactors from, and negate the *in vivo* function of, FsphDNase in the fungal/host interaction and therein to block phytoalexin production. However, EDTA applied at certain concentrations, can enhance the production of phytoalexin. An assay was developed [6] using agarose gel electrophoresis for DNA damage analysis that detects DNA alterations resulting from a component’s direct action on the DNA molecule. This technique offers an assay for assessing EDTA-caused DNA fragmentation. DNA fragmentation is temporally associated with early changes in the host chromatin [7]. Some of the chromatin changes have been shown to be the result of the ubiquitination of proteins (histones H2A/H2B and the transcription factor, HMG A) affecting regions containing PR genes. In addition to pathogen-derived-elicitors, the pea tissue responds to an extensive array of abiotic defense gene inducers [4] that possess defined actions within many biological systems. EDTA is now one that is of interest as a cellular DNA targeting compound.

### Molecular and Cytological Observations of the Pea Immune Response

The use of the pea pod endocarp model system allows an examination of synchronous molecular events as the elicitor applications rapidly contact all of the cells within the epidermal surface layer. The action of successful elicitors culminates in resistance that within 6 h completely suppresses further growth of a true, compatible pea pathogen [1] such as *Fusarium solani* f. sp*. pisi* (Fspi). If a functional induction of resistance is unsuccessful, the endocarp tissue hosts a susceptibility reaction that develops within 24 h. The mapping of regions within pea chromosomes revealed that some QTLs associated with disease resistance encompass map sites of pathogenesis-response (PR) genes [8,9].

The PR genes constitute the major components of disease resistance [10]. Thus, there are sensitive regions within pea chromatin associated with the pea disease resistance response. The induction and biosynthesis of the anti-fungal phytoalexin pisatin, as an isoflavonoid, requires the participation of multiple enzymes in this secondary pathway [11].

This suggests that the sensitive regions of pea chromatin itself may often serve as the initial target of the elicitors. Such a general action would support the prospect of a target being able simultaneously to activate genes located at multiple sites throughout the genome. This report on EDTA is designed to examine if its effect on pisatin accumulation, DNA damage, defense gene induction and cytological changes observed in relationship to the expression of disease resistance, may also be associated with a DNA target.

## 2. Results and Discussion

### 2.1. Phytoalexin Induction by EDTA

Table 1 indicates the concentrates of EDTA that affect the induction of the isoflavonoid phytoalexin, pisatin within 24 h after treatment. Pisatin as a phytoalexin possesses some antifungal properties. Concentrations as low as 7.8 mM EDTA independently induce pisatin accumulations. This concentration synergistically increases pisatin accumulation above that by the Fsph spores alone, e.g., {7.8 mM EDTA + spores treatment = 474 µg − (spores only treatment = 221 µg) = 85 µg/g fresh wt.}.

**Table 1:** The effect of a broad range of EDTA concentrations on the induction of pisatin accumulations in pea endocarp tissue in the presence and absence of *Fusarium solani* f. sp. *phaseoli* (Fsph) a pathogen of bean.

Treatment ^a^	Conc. EDTA mM	Pisatin μg/g frs.wt.
Water	0	0.0
EDTA	250	0.0
EDTA	125	12.4 ± 9.7
EDTA	62.5	62.2 ± 22.2
EDTA	31.2	194.2 ± 53.7
EDTA	15.6	136.2 ± 5.5
EDTA	7.8	85.8 ± 19.3
EDTA	3.9	3.3 ± 2.9
EDTA	1.9	0.6 ± 0.5
EDTA	0.9	0.0 ± 0.0
Water + Fsph spores	0.0	221.5 ± 67.9
EDTA + Fsph spores	250	1.7 ± 1.7
EDTA + Fsph spores	125	15.1 ± 2.1
EDTA + Fsph spores	62.5	86.3 ± 48.3
EDTA + Fsph spores	31.2	292.2 ± 115.2
EDTA + Fsph spores	15.6	428.9 ± 29.5
EDTA + Fsph spores	7.8	474.8 ± 16.3
EDTA + Fsph spores	3.9	353.6 ± 3.1
EDTA + Fsph spores	1.9	376.9 ± 39.7
EDTA + Fsph spores	0.9	258.0 ± 53.0

Notes: ^a^ Twenty μL of the indicated treatments were applied per pea pod half (~180 mg fresh weight) followed by 5 μL suspension of *F. solani* f. sp*. phaseoli* (Fsph) macroconidia 1.7 × 10^7^ spores/mL within 20 min (where indicated). Following a 24 h incubation period at 22 °C the pisatin was extracted overnight in 5 mL hexanes. Pisatin was quantified at 309 nm in ethanol.

### 2.2. Effect of EDTA on the Defense Response of Pea; Defense Gene Activation

In addition to the induced accumulation of pisatin, defense gene induction is often correlated with the actual suppression of the growth of a pathogen on peas [1]. However, resistance reportedly occurs as a result of the induction of multiple defense responses [10]. The gene products of the defense genes often referred to as “pathogenesis related” or PR proteins. Some of the genes code for enzymes on the pathway to pisatin production.

Primers were constructed (Table 2) to detect the activation of early expressed PR genes [12] coding for an array of functions: *DRR206* codes for an enzyme associated with a secondary pathway toward lignin (lignan) production. The pea gene *DRR230* coding for a defensin [13] has been established as resistance-conferring trait with defined antimicrobial activity [14]; *DRR49* (*PR-10*) codes for a product that enters the nucleus [15] and is putative RNase. *DRR49* trans-genetically confers resistance in potato to early blight [16]. The *PR1b* gene in *Arabidopsis* has a PR-1 function and is a “non-expressor” of *NPR1* which reportedly is a master, positive regulator of plant immunity in *Arabidopsis* [17]. NPR1 binds directly to salicylic acid (SA) and works as a SA receptor. In the presence and accumulation of SA [18], the NPR1 is reduced, monomerized and translocated to the nucleus. In the nucleus NPR1 interacts with the TGACG motif binding factor that binds to elements of the PR1 promoter. As a result, NPR1 is proposed to up-regulate a set of disease resistance genes via this route.

**Table 2:** Primers selected for real-time PCR analysis of pea PR genes.

Target	Genbank #	Real-time F primer	Real-time R primer
Pea Ubiquitin	L881142	GGCTAAGATACAGGACAAGGAG	AACGAAGGACAAGATGAAGGG
Pea Actin (Pea-ACT)	U81046	CACAATTGGCGCTGAAAGATT	GATCATCGATGGCTGGAACA
*DRR206*	U11716	CTTGGCTTAGTTTCACATTTGTTCTT	GGGTCAGCTCCAGCAAAAGTAA
*DRR230* (defensin)	L01579	TGTGGTGACAGAGGCAAACAC	TCGTGAAGCATACTCCCCTGTA
PR10 (AKA *DRR49*)	U31669	GATCTCATTCGAGGCTAAACTGTCT	CACACTCAGCTTTGCAATGGA
*PR1b*	AJ586324.1	AACTCATGTGCTGCTGGTTATCA	AACCGAATTGCGCCAAAC

**Figure 2:**
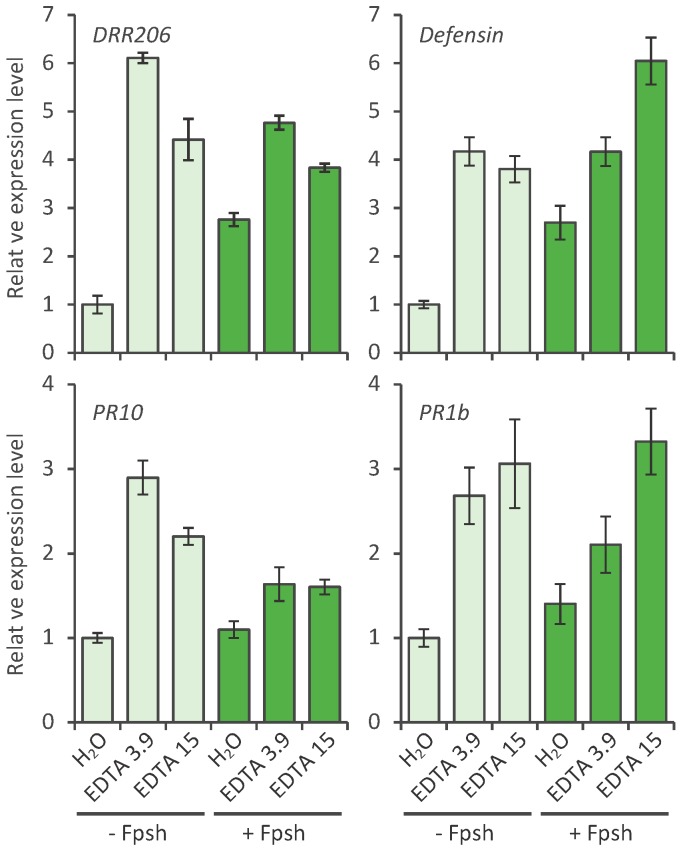
Effect of EDTA treatment on gene expression levels of pea pathogenesis-related genes. Pea endocarp tissues (0.4 g) were treated (20 µL/pod half) for 5 h with water (H_2_O), EDTA 3.9 mM, and EDTA 15 mM and after 20 min with (or without) 5 µL fungal spores (1.3 × 10^6^ spores/mL) of, *Fusarium solani* f. sp. *phaseoli* (Fsph). The tissues were then subjected to the qRT-PCR analysis. Histograms show the expression levels of pea pathogenesis-related genes. Data were normalized by the reference gene *Ubiquitin* and converted into a value relative to that of the water treatment control (-Fsph). Error bars represent standard error.

The data in Figure 2 indicate that EDTA concentrations of 3.9 and 15 mM effectively activate the PR genes: *DRR206*, the defensing, DRR230, *DRR49* (*PR10*) and *PR1b*, to levels above the water control. The 5 h activation level represents a stage 1 h prior to 6 h, a time point when nonhost resistance is maximal. The additive or synergistic enhancement of the induction of EDTA/Fsph spores treatments observed with pisatin accumulations was not consistently obtained in the induction of PR genes.

### 2.3. Direct Effect of EDTA on Fungal DNase

**Figure 3:**
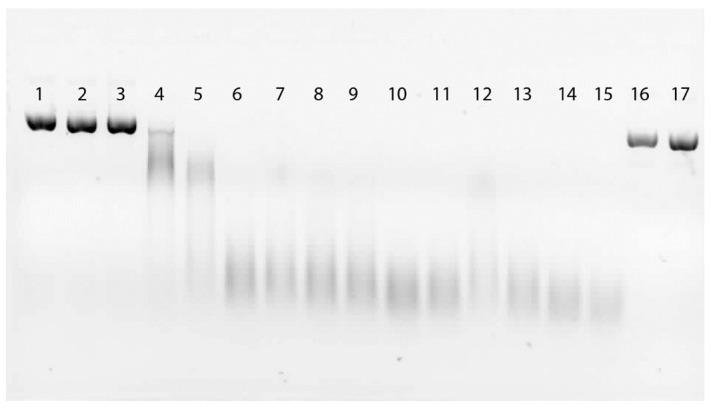
Effects of varying concentrations of EDTA on the enzymatic degradation of DNA. Each of the reactions loaded (1–15) contained 3 µL of fungal (*Verticillium dahlia*) DNase (2 Units), 2.2 µg plasmid DNA (in 10 mM MES pH 6.0) and 1 µL of the EDTA concentrations from 50 mM EDTA through half-fold dilutions to 0.003 mM EDTA. Reaction time was 10 min at 22 °C. Lanes 1, 2 and 3 with 50, 25 and 12 mM concentrations of EDTA, respectively, completely blocked DNA degradation. Treatments of 6.25 mM through 0.003 mM (Lanes 4–15) demonstrate the diminishing ability of EDTA to inhibit the DNase enzyme. Lanes 16 and 17 represent DNA controls without enzyme or EDTA.

As expected 50 mM EDTA directly inhibits *in vitro in vitro* activity of a fungal DNase expectantly by the chelation of Mn^2+^, a metal cofactor required for activity [2] ( Figure 3). These results suggest that the inhibition of a fungal DNase enzyme by EDTA does occur *in vitro*. However, this suppression is apparently not sufficient *in vivo* to block the phytoalexin-induction-potential of the fungus. Rather it appears that *in vivo* EDTA acted both independently and synergistically with the total eliciting potential of the fungus.

### 2.4. DNA Damage by EDTA

**Figure 4:**
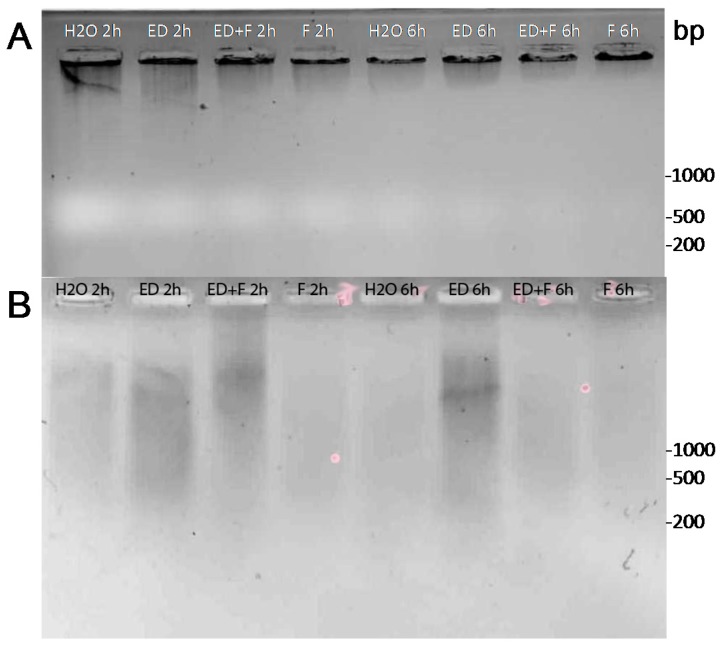
EDTA applications to pea endocarp tissue result in fragmentation of DNA in the period disease resistance is initiated (1–6 h). DNA was extracted from pea endocarp tissue (**A**) treated for 2 h and 6 h with (10 μL) water (H_2_O), 6 mM EDTA (ED), 6 mM EDTA + *Fusarium solani* f. sp. *phaseoli* spores (ED + F) or *F. solani* spores only (F). DNA (2 µg of each treatment was loaded per well) was separated on 1% agarose gels. The fragmented DNA was further separated from the bulk of the high molecular pea DNA (25 µg) by agarose retention disks under alkaline conditions (**B**) and the diffused fragmented DNA from the agarose disk recovered and separated on standard agarose gels. (Photos are inverted images of ethidium bromide stained DNA).

It was of interest to determine if direct DNA damage is a part of the EDTA action. The pea endocarp tissue was treated with EDTA only, EDTA with fungal spores, and fungal spores only (Figure 4). The extraction of pea genomic DNA from treated tissue represents primarily high molecular weight molecules. Therefore the detection of subtle single strand nicks requires that the resultant fragments in gel separations must occur under alkaline conditions that will allow the nicked DNA fragment to dis-engage as a single-stranded entity (Figure 4A). To further dis-engage the small fragments from the predominance of genomic DNA, the total DNA was trapped in a CHEF gel-type agar under alkaline conditions. The smaller single stranded fragments are allowed to diffuse out into an alkaline buffer over 48 h with slow stirring (Figure 4B). Gel separations of the total DNA aliquots reveal marginal effects of fragmentation associated with the EDTA treatments. Further separations of alkaline processed-and-released-DNA fragments reveal greater contrasts between EDTA-induced damage and control tissue. There are at least two confounding actions likely to be associated with the 2 and 6 h sampling times. First DNA repair is a rapid process in eukaryotic tissue and secondly some natural DNA fragmentation is present in excised water treated tissue. However the greater intensity of EDTA-induced DNA fragment is repeatedly observed.

### 2.5. Nonhost Resistance Induced by EDTA

**Figure 5:**
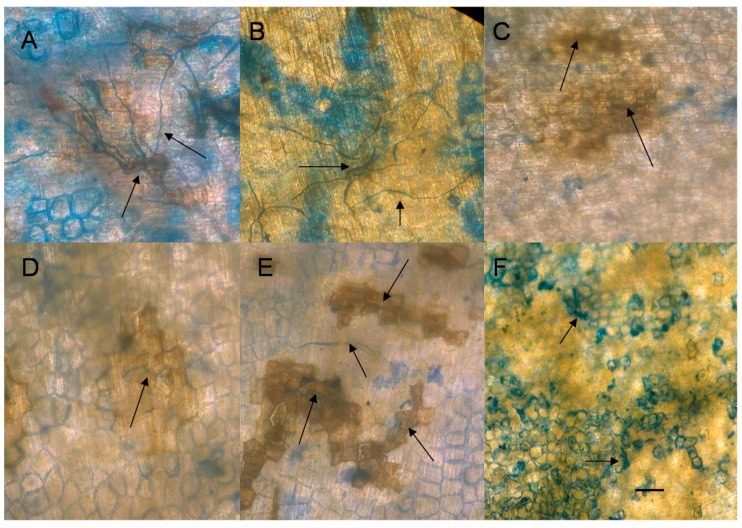
Low concentrations of EDTA induce resistance in pea endocarp tissue against the pea pathogen, *Fusarium solani* f. sp. *pisi* (Fspi). Twenty min of pretreatments (10 μL) of (**A**) water and (**B**) 6 mM EDTA; (**C**) 3 mM EDTA; (**D**) 1.5 mM EDTA; (**E**) 0.7 mM; and (**F**) 0.3 mM EDTA concentrations were applied to pea tissue 20 min prior to 5 µL of a 1.3 × 10^6^ spore suspension of Fspi. Photos represent the extent of growth typical from the lesion observations of 30 spores for each concentration of EDTA applied. Arrows indicate the location of the spores or mycelia distorted or suppressed by the treatments. (Note: Bar = 50 microns.)

The effect of EDTA applied 20 min prior to the inoculation with spores of the true pea pathogen, *Fusarium solani* f. sp. *pisi* (Fspi) is concentration dependent. EDTA at 6 mM effectively broke resistance whereas EDTA applied at 3–0.3 mM effectively promoted complete resistance against the pathogen (Figure 5).

### 2.6. Cytological Effect of EDTA on Pea Nuclear Condition

**Figure 6:**
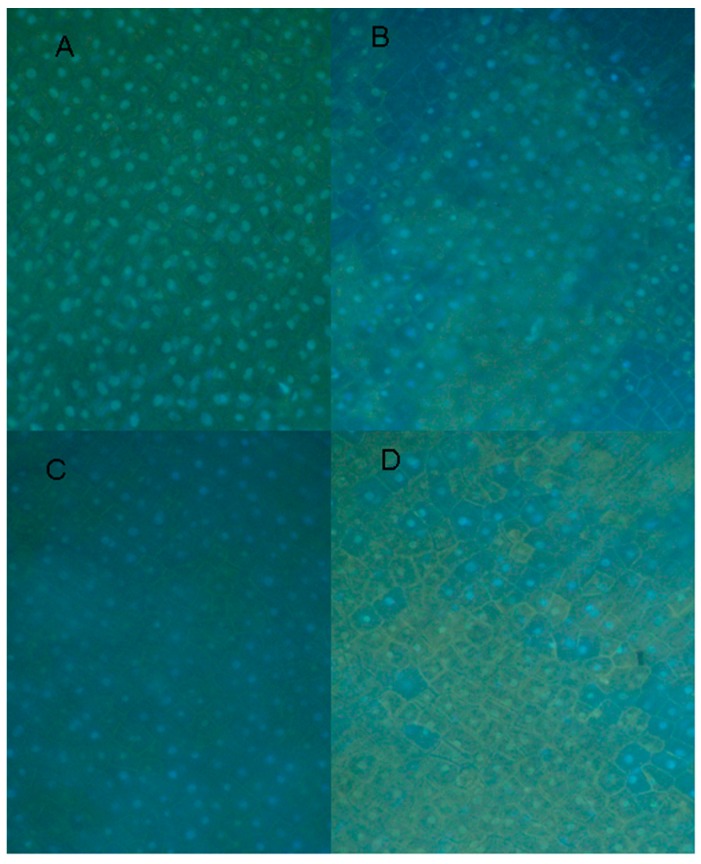
Effect of EDTA on the condition of nuclei within pea endocarp tissue. Pea endocarp tissue was treated 3 h with (**A**) water, (**B**) 12 mM EDTA, (**C**) 6mM EDTA or (**D**) 3 mM EDTA. Surface cell sections were treated with DAPI and photographed via a fluorescence microscope. Nuclei (average of 40 visible nuclei remaining intact) measured 10.0, 7.94, 9.74 and 8.7 microns, respectively, adjusted proportionally to the normal average of 10 microns previously determined by electron microscope analysis in control tissue.

The exposed endocarp surface cells of a split pea pod are without a cuticle layer and thus can be DNA-stained with DAPI without fixing and the condition of a large number of the nuclei in the surface cell layer can be determined via a fluorescent microscope. The photographs of Figure 6 indicate nuclear changes and the diminishing detection of DAPI staining. The sizing of the visible nuclei in tissue treated 3 h with 12, 6 and 3 mM EDTA indicates slight but significant reductions in size from those in water treated tissue. The effects that caused the diminished detection of the DAPI stain are unknown.

## 3. Experimental Section

### 3.1. Plant and Pathogens

Immature pea pods (2 cm in length) were harvested from *Pisum sativum* cv. Lance plants grown in sand in the greenhouse (12 h light). All fungi were cultured on potato dextrose agar (PDA) (Difco) supplemented with pea pods (5 g/L). Fungal cultures used in this study were *Fusarium solani* f. sp*. pisi* Snyder & Hansen (ATCC No. 38136) (Fspi) and *F. solani* f. sp. *phaseoli* Snyder & Hansen (ATCC No. 38135) (Fsph) from pea and bean respectively. Cell death was assayed by staining plant cells with 1 mg/mL trypan blue.

### 3.2. EDTA Treatment and Pisatin Quantization

Immature pea pods (2 cm) were separated into halves with a smooth spatula. Treatments (20 µL/pod half) were applied and distributed to the exposed endocarp surface. Following 24 h at 100% humidity and 22 °C the pod halves were submerged into 5 mL hexanes for 24 h. The hexanes were volatilized away and the residual material containing pisatin was dissolved in 95% ethanol and quantitated at UV 309 nm in a spectrophotometer.

### 3.3. Activation of PR Genes

Procedures for total RNA isolation and purification were briefly described as follows: pea endocarp tissues (pod halves) were treated the designated treatment and after 5 ½ h the tissues were ground in liquid nitrogen and solubilized in the extraction buffer, (sodium perchlorate, 5 M; Tris base, 0.5 M; sodium dodecyl sulfate, 2.5%; NaCl, 0.05%; and disodium-EGTA, 0.05 M). The nucleic acids were precipitated from the aqueous phase of a chloroform/phenol extraction and then dissolved in water. The nucleic acid solution was adjusted to 2.0 M lithium chloride to precipitate the RNA. The total RNA from each sampled was subjected to quantitative real-time reverse transcription-polymerase chain reaction (qRT-PCR) using the CFX96 system (Bio-Rad Laboratories, Inc., Hercules, CA, USA). The primers used are described in Table 2.

## 4. Conclusions

The defense response of pea endocarp tissue to pathogen challenge or to chemical elicitors appears to depend on both the chemistry of the eliciting compound and of the cellular target. Although EDTA is most known as a chelating agent it has the potential to alter the DNA of pea nuclei. This potential was realized in the absence of EDTA-related cell death (data not shown). Further the concentrations of EDTA active in inducing resistance in pea to the pea pathogen, Fsph, were not effective in directly inhibiting fungal growth in liquid culture (data not shown). Interestingly a structurally similar chelator [19], EGTA, was not effective in inducing pisatin accumulations (data not shown) Previous research on the pea/Fsph model for studying non-host disease resistance has indicated that chromatin can be a primary target for activating the defense genes [7,12]. These PR (pathogenesis-related) genes are distributed in various regions within the pea genome [8,9,10]. Other eukaryotic research has revealed the numerous ways that RNA polymerase complexes, that are stalled upstream of some genes, can be released for transcription by helical changes in the DNA or the substitution or removal of nuclear proteins [20,21]. In the defense response of pea tissue the nuclear proteins HMG A and histones H2A and H2B are reduced by ubiquitination [7]. EDTA at low concentrations appears capable of causing some of these changes through its action of subtly fragmenting pea DNA. Applications of EDTA to corneal epithelial cells also increases DNA single and double stranded breaks [22] and were proposed to be caused indirectly by way of the reactive oxygen species of oxidative stress. The EDTA-related mechanism for activating defense genes in peas appears to be directly on the DNA, since an extensive screening of DNA-specific components with known actions, e.g., intercalation, minor groove insertion, helical alteration, DNase action and thymidine dimerization are also capable of activating PR genes and/or phytoalexin accumulations [4,23,24,25].

Pathologists are in a continual search for components that can activate defense responses in the absence of major detrimental side effects and major costs. Thus there appear to be future practical applications of these EDTA properties in agriculture.
